# Association between initial ventilation mode and hospital outcomes for severe congenital diaphragmatic hernia

**DOI:** 10.1038/s41372-024-02024-z

**Published:** 2024-06-28

**Authors:** K. Taylor Wild, Leny Mathew, Anne M. Ades, Natalie E. Rintoul, Leane Soorikian, Kelle Matthews, Sura Lee, K. Taylor Van Hoose, Erin Kesler, Sabrina Flohr, Anna Bostwick, Tom Reynolds, Holly L. Hedrick, Elizabeth E. Foglia

**Affiliations:** 1 Division of Neonatology, Children’s Hospital of Philadelphia, Perelman School of Medicine at University of Pennsylvania, Philadelphia, PA USA; 2 Center for Fetal Diagnosis and Treatment, Children’s Hospital of Philadelphia, Philadelphia, PA USA; 3 Division of Pediatric General, Thoracic, and Fetal Surgery, Children’s Hospital of Philadelphia, Philadelphia, PA USA

**Keywords:** Outcomes research, Paediatrics

## Abstract

**Objective:**

To determine the association between initial delivery room (DR) ventilator (conventional mechanical ventilation [CMV] versus high frequency oscillatory ventilation [HFOV] and hospital outcomes for infants with severe congenital diaphragmatic hernia (CDH).

**Study design:**

Quasi-experimental design before/after introducing a clinical protocol promoting HFOV. The primary outcome was first blood gas parameters. Secondary outcomes included serial blood gas assessments, ECMO, survival, duration of ventilation, and length of hospitalization.

**Results:**

First pH and CO_2_ were more favorable in the HFOV group (*n* = 75) than CMV group (*n* = 85), median (interquartile range (IQR)) pH 7.18 (7.03, 7.24) vs. 7.05 (6.93, 7.17), adjusted *p*-value < 0.001; median CO_2_ 62.0 (46.0, 82.0) vs 85.9 (59.0, 103.0), adjusted *p*-value < 0.001. ECMO, survival, duration of ventilation, and length of hospitalization did not differ between groups in adjusted analysis.

**Conclusion:**

Among infants with severe CDH, initial DR HFOV was associated with improved early gas exchange with no adverse differences in hospital outcomes.

## Introduction

Congenital diaphragmatic hernia (CDH) occurs when the fetal abdominal viscera herniate into the chest through a diaphragmatic defect. While pulmonary hypoplasia and pulmonary hypertension are the main causes of mortality in infants with CDH, ventilator-induced lung injury and oxygen toxicity are responsible for significant morbidity and may result in prolonged oxygen dependency [1]. Ventilator-induced lung injury begins with the first exposure to positive pressure ventilation, thus early initiation of lung protective strategies is critical. Compared with conventional mechanical ventilation (CMV), high frequency oscillatory ventilation (HFOV) may improve gas exchange at lower mean airway pressures thus reducing the risk of barotrauma. As hypercarbia in the first hours of life is associated with mortality and extracorporeal membrane oxygenation (ECMO) in infants with CDH, preventing early hypercarbia may be important in reducing these undesired outcomes [2–7].

A randomized clinical trial of CMV versus HFOV for CDH, known as the VICI trial, randomized infants with CDH to CMV versus HFOV within one hour of delivery. There was no difference in the primary composite outcome of mortality or chronic lung disease, but infants in the CMV group experienced shorter duration of ventilation and reduced need for ECMO [8, 9]. However, the VICI trial used relatively high HFOV mean airway pressure settings, raising questions about whether the VICI trial results apply to other delivery room settings.

We hypothesized that initial HFOV in the delivery room (DR) would be associated with improved measures of early gas exchange. In 2019, the Children’s Hospital of Philadelphia (CHOP) implemented a practice change of initiating ventilatory support with HFOV as the initial ventilator mode in the DR for infants with severe CDH. This study sought to determine whether initial mode of ventilation (CMV vs HFOV) in the DR for infants with severe CDH is associated with measures of early gas exchange, and whether initial mode of ventilation (CMV vs HFOV) for infants with severe CDH is associated with hospital-based outcomes.

## Methods

### Design

Quasi-experimental design before/after introducing a new clinical protocol for infants with severe CDH. From 2014 to 2018, all infants with CDH were intubated immediately after birth and placed on CMV. A new protocol was introduced in 2019 that specified that all infants with severe CDH, defined as an intrathoracic liver or a severe right sided CDH, are transitioned to HFOV immediately after intubation. We excluded infants born prior to 2014 to ensure other aspects of resuscitation were consistent between groups. Otherwise, we included all infants with an intrathoracic liver or a right sided CDH who received active treatment and who were treated per the assigned protocol per epoch. The CHOP Institutional Review Board approved this study (IRB 21-018553) with a waiver of informed parental consent.

### Hospital resuscitation protocol

Per protocol, all infants with CDH are intubated immediately after birth, and intermittent positive pressure ventilation (PPV) with a T-piece ventilator is initiated with peak inspiratory pressures (PIPs) of 20–25 cm H_2_O, positive end expiratory pressure (PEEP) of 5 cm H_2_O, and a ventilation rate of 40–50 breaths/min. For infants managed with CMV in the 2014–2018 era, initial recommended CMV settings were PIP 20–25 cm H_2_O, PEEP 5 cm H_2_O, and ventilation rate 40–50 breaths/min. Since 2019, the DR protocol recommends that all infants with severe CDH are transitioned to HFOV after the endotracheal tube is secured. Recommended HFOV settings include Mean Airway Pressure (MAP) of 11–13 cm H_2_O, amplitude of 30–35 cm H_2_O that is adjusted to achieve appropriate chest wall vibration, and frequency or Hertz (Hz) of 6 for term infants that is increased with decreasing gestational age. Recommended initial FiO_2_ is 50% (this was consistent in both eras). FiO_2_ is subsequently titrated to achieve pre-ductal oxygen saturation goals of ≥85% by 10 min of life and beyond. Following intubation, gastric decompression, and placement on the assigned ventilator, umbilical lines are placed, and a post-ductal arterial blood gas is obtained. DR resuscitation and stabilization typically takes about one hour and the first blood gas is obtained in the DR while on the initial mode of ventilation. Infants are then transferred to the NICU. The DR and NICU are 4 floors apart in the same wing of the hospital with a transport time of approximately 5–10 min. Infants on HFOV are briefly placed on CMV to complete this short transport from the DR to the NICU.

### Exposure of interest

The exposure of interest was mode of ventilation in the DR and year of birth. We included all infants born before the policy change (birth years 2014–2018) who were managed with DR CMV in the CMV cohort. We included all infants born after the policy change (birth years 2019–2022) managed with DR HFOV in the HFOV cohort. Infants who were not treated per protocol in either era were excluded.

### Main outcome measures

The primary outcome was blood gas parameters on the first blood gas obtained in the DR after the infant was placed on the protocolized ventilator mode. Secondary outcomes included serial blood gas assessments throughout the first 48 h of life, ECMO, survival, duration of invasive ventilation, duration of non-invasive ventilation, and length of hospital stay.

### Data analysis

Unadjusted analyses were conducted with Wilcoxon rank-sum test for continuous variables and chi-squared test or the Fisher’s exact test, as appropriate, for categorical variables. The association between ventilator mode and outcomes of interest was assessed using logistic regression for ECMO and survival to discharge and linear regression for blood gas parameters, duration of ventilation, and length of hospital stay. All regression models included terms for CDH side and O/E LHR to adjust for the severity of CDH. For the purpose of this analysis, the anteroposterior (AP) method was used for O/E LHR as it was consistently available for all years of this cohort. The models for initial blood gas additionally included time to the first blood gas (relative to birth) to adjust for potential differences in time to blood gas between the two modes of ventilation. Of note, blood gases obtained for infants on ECMO were excluded from this analysis as blood gas values would then reflect the ECMO circuit. The models for length of stay and duration of ventilation were estimated only among survivors of the neonatal intensive care unit (NICU) hospitalization. Linear regression models for length of stay and duration of ventilation were repeated using natural log transformed outcome variables as a sensitivity analysis assessing deviations from the normality assumption. All analyses were conducted in R V.4.1.2.

## Results

**Fig. 1:**
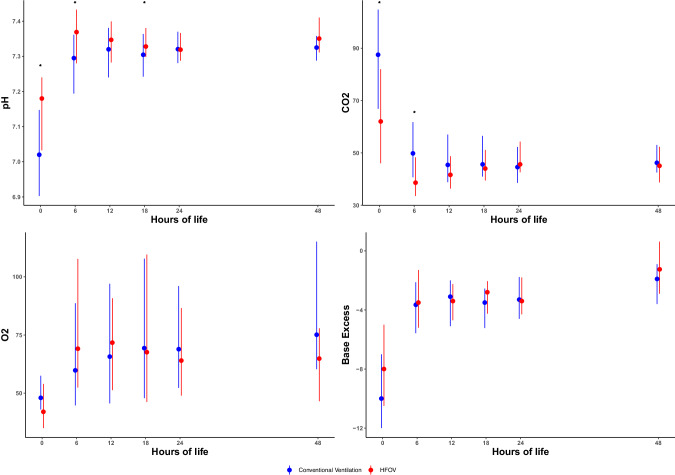
Blood gas values are shown for the first 48 h of life. Infants who were on CMV in the delivery room are shown in blue and infants who were on HFOV in the delivery room are shown in red. As noted by *, pH was significantly higher and CO_2_ significantly lower for the first 6 h of life in the HFOV group. Of note, once placed on ECMO infants were excluded from this analysis as blood gas values would then reflect the ECMO circuit.

There were 85 and 75 infants with severe CDH treated per assigned protocol with CMV and HFOV, respectively (Table 1). The cohort flow diagram is displayed in Supplementary Fig. 1. Of note, infants in the HFOV group had a significantly lower Observed to Expected Lung to Head Ratio (O/E LHR), median (interquartile range), 26.5 (22.0, 34.5) versus 38.0 (27.9, 47.5); *p* < 0.001. There were no differences in delivery room characteristics (Table 2). Infants were transitioned to HFOV by a median of 10 min of life compared to infants in the CMV group who received intermittent PPV by 3 min of life. There were no infants initially managed with HFOV and then transitioned to CMV in the DR. Of the 75 infants managed with HFOV in the DR, 68 (91%) remained on HFOV upon NICU admission, and the remaining 7 (9%) were transitioned to CMV upon NICU admission. Among 85 infants initially managed with CMV, 21 (25%) remained on CMV upon NICU admission, and the remaining 64 (75%) were transitioned to HFOV upon admission. The decision to transition to HFOV after NICU admission was made by the attending neonatologist in response to postnatal markers of gas exchange (i.e., initial blood gas results and FiO_2_ requirements). First blood gas was obtained in the DR while on the initial mode of ventilation and the assigned ventilation cohort in this study. Initial blood gas demonstrated higher pH and lower CO_2_ in the HFOV group compared with CMV, which was also observed at 6 h after birth. Beyond six hours there were intermittent differences in gas parameters that were statistically but not clinically significant between groups (Fig. 1, Supplementary Table 1). ECMO use was higher in the HFOV group in the unadjusted analysis, but this result was not statistically significant after adjusting for CDH side and O/E LHR. In adjusted analysis there were no differences in survival, duration of invasive or non-invasive ventilation days, or length of stay among survivors (Table 3). There were no differences in the use of inhaled nitric oxide, dopamine infusions, or use of pulmonary hypertension medications at discharge, however there was increased use of epinephrine and alprostadil in the more contemporary HFOV cohort during the NICU hospitalization following the initial delivery room stabilization (Supplementary Table 2).

**Table 1:** Cohort characteristics.

Characteristic	*n*(%), Mean ± SD, Median (IQR)		*p*-value
	CMV (*n* = 85)	HFOV (*n* = 75)	
Sex (Male)	51 (60.0%)	41 (54.7%)	0.60
O/E LHR^a^ (%)	38.0 (27.9, 47.5)	26.5 (22.0, 34.5)	**<0.001**
Gestational age at time of prenatal imaging (weeks)	23.0 (22.0, 27.1)	23.5 (22.1, 26.4)	0.55
Mode of delivery (Vaginal)	47 (55.3%)	35 (46.7%)	0.35
Gestational age at birth (weeks)	38.8 (37.9, 39.4)	38.6 (37.2, 39.3)	0.24
Birthweight (kg)	3.03 (2.80, 3.40)	2.80 (2.52, 3.27)	0.01

*CDH* Congenital Diaphragmatic Hernia, *CMV* Conventional Mechanical Ventilation, *HFOV* High Frequency Oscillatory Ventilation, *IQR* interquartile range, *LHR* Lung to Head Ratio, *O/E* Observed to Expected LHR, *SD* standard deviation.

Bold values indicate statistical significance.

^a^Observed to Expected (O/E) Lung to Head Ratio (LHR) was measured by mid trimester ultrasound using the AP method of the TOTAL trial [17].

**Table 2:** Delivery room characteristics.

Characteristic	*n*(%), Median (IQR)		*p*-value
	CMV (*n* = 85)	HFOV (*n* = 75)	
1 min Apgar	5 (2, 7)	5 (4, 7)	0.13
5 min Apgar	8 (6, 9)	8 (6, 8)	0.83
Time to Pre-ductal SpO_2_ > 85% (min)	11.0 (7.0, 17.8)	10.5 (7.0, 21.3)	0.68
Time to HR > 100 beats per min (min)	3.0 (1.0, 5.0)	3.0 (2.0, 6.0)	0.17
Last FiO_2_ in delivery room	80 (50, 100)	80 (50, 100)	0.75
Fentanyl given in delivery room	65 (76.5)	61 (81.3)	0.56
Vecuronium given in delivery room	17 (20.0)	37 (49.3)	**0.001**
Normal saline bolus given in delivery room	38 (44.7)	34 (45.3)	0.99
Time placed on ventilator after birth (min)	3.0 (2.0, 5.0)	10.0 (7.0, 18.0)	**<0.001**
Time in delivery room (min)	60.0 (53.0, 74.0)	65.0 (59.0, 83.0)	0.003

Ventilator settings in the delivery room			
CMV		HFOV	
PIP (cm H_2_O)	25 (20, 25)	MAP (cm H_2_O)	13 (12, 13)
PEEP (cm H_2_O)	5 (5, 6)	Amplitude (cm H_2_O)	33 (30, 36)
Rate (breaths/min	50 (40, 60)	Frequency (Hz)	7 (6, 8)

Bold values indicate statistical significance.

**Table 3:** Association between initial ventilator mode and outcomes.

	CMV (*n* = 85)	HFOV (*n* = 75)	Unadjusted model		Adjusted model^a^
	Median (IQR)		Linear regression		
First blood gas					
Time to first blood gas (min)	40.0 (34.0, 47.0)	44.5 (38.0, 52.0)	Coefficient (95% CI)	Coefficient (95% CI)	
pH	7.02 (6.90, 7.15)	7.18 (7.04, 7.24)	0.12 (0.06, 0.18)	**0.17** (**0.10, 0.23)**	
CO_2_	87.5 (66.9, 105.0)	62.0 (46.0, 82.5)	**−21.24** **(−30.12, −12.37)**	**−28.49** **(−38.18, −18.80)**	
PaO_2_	48.0 (43.0, 57.5)	42.0 (35.3, 54.0)	−3.75 (−13.09, 5.60)	2.60 (−7.84, 13.03)	
Base deficit	−10.0 (−12.0, −7.0)	−8.0 (−10.3, −5.0)	1.42 (**−**0.15, 3.00)	**2.49** **(0.68, 4.29)**	
Duration of invasive ventilation among survivors (days)	36.5 (22.5, 57.5)	36.0 (25.0, 59.0)	17.00 (−6.56, 40.56)	8.64 (−17.59, 34.88)	
Duration of non-invasive ventilation among survivors (days)	23.0 (11.5, 52.5)	37.0 (22.0, 94.0)	12.85 (−18.14, 43.84)	−7.89 (−40.41, 24.62)	
Length of stay among survivors (days)	79.1 (55.4, 132.0)	85.0 (63.4, 172.0)	21.64 (−10.63, 53.90)	0.01 (−34.47, 34.47)	

			Logistic regression		
	*n*(%)		Odds ratio (95% CI)	Odds ratio (95% CI)	
ECMO	25 (29.4%)	36 (48.0%)	2.22 (1.16, 4.28)	1.45 (0.71, 2.98)	
Survival to discharge	65 (76.5%)	58 (77.3%)	0.95 (0.45, 1.99)	1.00 (0.44, 2.30)	

^a^All regression models were adjusted for CDH side and O/E LHR. Models for blood gas measures were additionally adjusted for time to first blood gas.

Bold values indicate statistical significance.

## Discussion

Infants with severe CDH are intubated immediately after birth [10, 11], but the optimal initial mode of ventilation remains unclear for this population. In this study, we leveraged our high-volume population of infants with severe CDH to address this evidence gap. In our experience, HFOV as the initial mode of ventilation in the DR for infants with severe CDH was associated with improved gas exchange during the postnatal transition without observed differences in adverse hospital outcomes.

The existing literature for initial mode of ventilation for infants with CDH is inconsistent. The VICI trial randomized 91 infants with CDH to CMV compared to 80 infants to HFOV. While there was no significant difference in the combined outcome of mortality or chronic lung disease, infants randomized to HFOV experienced higher ECMO use and longer duration of mechanical ventilation [8, 9]. Conversely, Fuyuki et al. conducted a multicenter retrospective cohort study of initial mode of ventilation within the first 24 h for 135 infants with CDH. Following propensity score matching of 45 infants treated with CMV and 90 with HFOV, there were no differences in outcomes of ECMO, mortality, or chronic lung disease [12]. Finally, Semama et al. compared outcomes of infants with CDH between 2 French centers; one center prioritized CMV and the other prioritized HFOV using low MAP < 12 cm H_2_O. Duration of oxygen therapy and survival did not differ between centers, but infants treated in the center that prioritized HFOV experienced longer duration of mechanical ventilation and sedation. Of note, sedation and hemodynamic management differed between the two centers [13].

In the present study, initial mode of ventilation was not associated with mortality, ECMO, or duration of ventilation after adjusting for CDH side and O/E LHR. A notable difference between the VICI trial and ours was CDH severity. In our study, the median O/E LHR was 26.5% in the HFOV group and 36.6% in the CMV group compared to 47% in the HFOV group and 48% in the CMV group in the VICI trial. This difference is important as mortality and ECMO utilization are known to be higher in infants with more severe CDH [7, 14, 15]. Despite the high severity in our cohort, overall mortality was similar to the CMV group in the VICI trial who had less severe CDH. Our more contemporary HFOV group also had more severe CDH. To account for this, models were adjusted for CDH side and O/E LHR.

Ventilator settings are another important component to consider, particularly the mean airway pressure and the balance between achieving lung inflation and avoiding overdistension once aerated. In the VICI trial, initial CMV settings were PIPs of 20–25 cm H_2_O and PEEP of 3–5 cm H_2_O, with a ventilator rate of 40–60/min. These are very similar to our CMV settings with the exception of PEEP, which was consistently set at 5–6 cm H_2_O at our center. In contrast, the HFOV settings used in our DR differed substantially from those used in the VICI trial. In the VICI trial, initial HFOV settings included a MAP 13–17 cm H_2_O, frequency of 10–12 Hz, and amplitude of 30–50 cm H_2_O depending on chest wall vibration. The MAP settings used for HFOV in our study were lower than those used in VICI; the median MAP was 13 cm H_2_O, frequency was 7 Hz, and amplitude was 33 cm H_2_O. We speculate that HFOV settings used in our cohort may be more lung protective than those used in the VICI trial. This is important as MAP settings below 13 cm H_2_O have been associated with lower ECMO, vasoactive drugs, and inhaled nitric oxide use [16].

Previous studies have demonstrated hypercarbia after birth to be associated with increased mortality and ECMO use [2–7]. In our study, HFOV was associated with improved gas exchange immediately after birth. Although this did not translate to benefit for ECMO or mortality, preventing severe acidosis may have a beneficial impact on cerebral hemodynamics, pulmonary hypertension, cardiac function, and neurodevelopment. Future areas of study may assess the impact of severe hypercarbia and acidosis on neurologic and cardiac outcomes among infants with CDH.

Our study has limitations and unique strengths. This was an observational cohort study and therefore a non-randomized sample. We adjusted for known confounders, such as CDH side and severity in our analysis. We chose to evaluate hospital based ventilatory outcomes as those were the outcomes reported in the VICI trial. We acknowledge that other important outcomes such as cardiac function, pulmonary hypertension, and other complications were not evaluated. In Supplementary Table 2, we compare vasoactive support and pulmonary hypertension treatment between the two groups. While there were no differences in the use of inhaled nitric oxide, dopamine infusions, or use of pulmonary hypertension medications at discharge, there was increased epinephrine and alprostadil use in the more contemporary HFOV cohort. Use of epinephrine infusions was intentionally increased in an effort to decrease use of high dose dopamine infusions. These differences reflect practice changes in our center that took place following DR resuscitation. Echo-guided hemodynamic management was not a standardized practice in our center across both epochs. We also did not report long term neurodevelopmental outcomes. In addition, we defined our cohort to ensure there were no other important changes in DR clinical practice during the study period. However, it is possible that unmeasured confounders differed between groups. As a single center study, outcomes may also reflect practice management differences unique to our center. Finally, we used a sample of convenience based on the number of infants treated with each ventilator during the study period; our sample size was similar to that of the VICI trial. It is possible that we were underpowered to detect differences in clinical outcomes between groups. Study strengths include one of the largest cohorts of infants with CDH comparing CMV and HFOV. In addition, we care for a large population of infants with severe CDH, and this study fills an important evidence gap specific to this high-risk population that is not always represented in clinical trials.

## Conclusion

HFOV as the initial mode of ventilation in the DR for infants with severe CDH was associated with improved gas exchange immediately after resuscitation without increased mortality, ECMO utilization, duration of ventilation, or length of stay. HFOV is an effective and safe mode of initial ventilation for infants with severe CDH.

## Supplementary information

Supplemental Figure 1. Flow diagram of cohort based on initial ventilator mode.

Supplemental Table 1. Blood Gas Values for the First 48 Hours of Life

Supplemental Table 2. Vasoactive Use and Pulmonary Hypertension Treatment

## Data Availability

The data that support the findings of this study are not publicly available due to privacy reasons but are available from the responding author upon reasonable request.

## References

[1] van den Hout L, Reiss I, Felix JF, Hop WCJ, Lally PA, Lally KP, et al. Risk factors for chronic lung disease and mortality in newborns with congenital diaphragmatic hernia. Neonatology. 2010;98:370–80. 21042035 10.1159/000316974

[2] Schultz CM, Digeronimo RJ, Yoder BA. Congenital diaphragmatic hernia: a simplified postnatal predictor of outcome. J Pediatr Surg. 2007;42:510–6. 17336189 10.1016/j.jpedsurg.2006.10.043

[3] Salas AA, Bhat R, Dabrowska K, Leadford A, Anderson S, Harmon CM, et al. The value of Pa(CO2) in relation to outcome in congenital diaphragmatic hernia. Am J Perinatol. 2014;31:939–46. 24515620 10.1055/s-0034-1368088

[4] Khmour AY, Konduri GG, Sato TT, Uhing MR, Basir MA. Role of admission gas exchange measurement in predicting congenital diaphragmatic hernia survival in the era of gentle ventilation. J Pediatr Surg. 2014;49:1197–201. 25092075 10.1016/j.jpedsurg.2014.03.011

[5] Abbas PI, Cass DL, Olutoye OO, Zamora IJ, Akimkuoto AC, Sheikh F, et al. Persistent hypercarbia after resuscitation is associated with increased mortality in congenital diaphragmatic hernia patients. J Pediatr Surg. 2015;50:739–43. 25783376 10.1016/j.jpedsurg.2015.02.028

[6] Patel MJ, Bell CS, Lally KP, Lally PA, Katakam LI. Lowest PaCO2 on the first day of life predicts mortality and morbidity among infants with congenital diaphragmatic hernia. J Perinatol. 2019;39:229–36. 30425337 10.1038/s41372-018-0269-6

[7] Kipfmueller F, Schroeder L, Melaku T, Geipel A, Berg C, Gembruch U, et al. Prediction of ECMO and Mortality in Neonates with Congenital Diaphragmatic Hernia Using the SNAP-II Score. Klin Padiatr. 2019;231:297–303. 31569261 10.1055/a-1009-6671

[8] van den Hout L, Tibboel D, Vijfhuize S, te Beest H, Hop W, Resiss I, et al. The VICI-trial: high frequency oscillation versus conventional mechanical ventilation in newborns with congenital diaphragmatic hernia: an international multicentre randomized controlled trial. BMC Pediatr. 2011;11:98. 22047542 10.1186/1471-2431-11-98 PMC3226543

[9] Snoek KG, Capolupo I, van Rosmalen J, de Jongste-van den Hout L, Vijfhuize S, Greenough A, et al. Conventional mechanical ventilation versus high-frequency oscillatory ventilation for congenital diaphragmatic hernia: a randomized clinical trial (The VICI-trial). Ann Surg. 2016;263:867–74. 26692079 10.1097/SLA.0000000000001533

[10] Snoek KG, Reiss IKM, Greenough A, Capolupo R, Urlesberger B, Wessel L, et al. Standardized Postnatal Management of Infants with Congenital Diaphragmatic Hernia in Europe: The CDH EURO Consortium Consensus - 2015 Update. Neonatology. 2016;110:66–74. 27077664 10.1159/000444210

[11] Jancelewicz T, Brindle ME, Guner YS, Lally PA, Lally KP, Harting MT. Toward standardized management of congenital diaphragmatic hernia: an analysis of practice guidelines. J Surg Res. 2019;243:229–35. 10.1016/j.jss.2019.05.007 31226462 10.1016/j.jss.2019.05.007

[12] Fuyuki M, Usui N, Taguchi T, Hayakawa M, Masumoto K, Kanamori Y, et al. Prognosis of conventional vs. high-frequency ventilation for congenital diaphragmatic hernia: a retrospective cohort study. J Perinatol. 2021;41:814–23. 33177680 10.1038/s41372-020-00833-6

[13] Semama C, Vu S, Kyheng M, Le Duc K, Plaisant F, Storme L, et al. High-frequency oscillatory ventilation versus conventional ventilation in the respiratory management of term neonates with a congenital diaphragmatic hernia: a retrospective cohort study. Eur J Pediatr. 2022;181:3899–906. 35994123 10.1007/s00431-022-04590-w

[14] Gupta VS, Harting MT, Lally PA, Miller CC, Hirschl RB, Davis CF, et al. Mortality in Congenital Diaphragmatic Hernia: A Multicenter Registry Study of Over 5000 Patients Over 25 Years. Ann Surg. 2021. 10.1097/SLA.0000000000005113. 10.1097/SLA.0000000000005113 34334632

[15] Guner YS, Delaplain PT, Zhang L, Di Nardo M, Brogan TV, Chen Y, et al. Trends in mortality and risk characteristics of congenital diaphragmatic hernia treated with extracorporeal membrane oxygenation. ASAIO. 2019;65:509–15. 10.1097/MAT.0000000000000834 10.1097/MAT.0000000000000834 PMC6251767 29863628

[16] Yang MJ, Fenton S, Russell K, Yost CC, Yoder BA. Left-sided congenital diaphragmatic hernia: can we improve survival while decreasing ECMO? J Perinatol. 2020;40:935–42. 32066841 10.1038/s41372-020-0615-3

[17] DeKoninck P, Gratacos E, Van Mieghem T, et al. Results of Fetal Endoscopic Tracheal Occlusion for congenital diaphragmatic hernia and the set up of the randomized controlled TOTAL trial. Early Human Development. 2011;87(9):619–624. 10.1016/j.earlhumdev.2011.08.001 21907109

