# Supplementary figures and images for: Association between initial ventilation mode and hospital outcomes for severe congenital diaphragmatic hernia
Source: J Perinatol. 2024 Jun 28;44(9):1353–8. doi: 10.1038/s41372-024-02024-z (PMC11379620; doi:10.1038/s41372-024-02024-z)

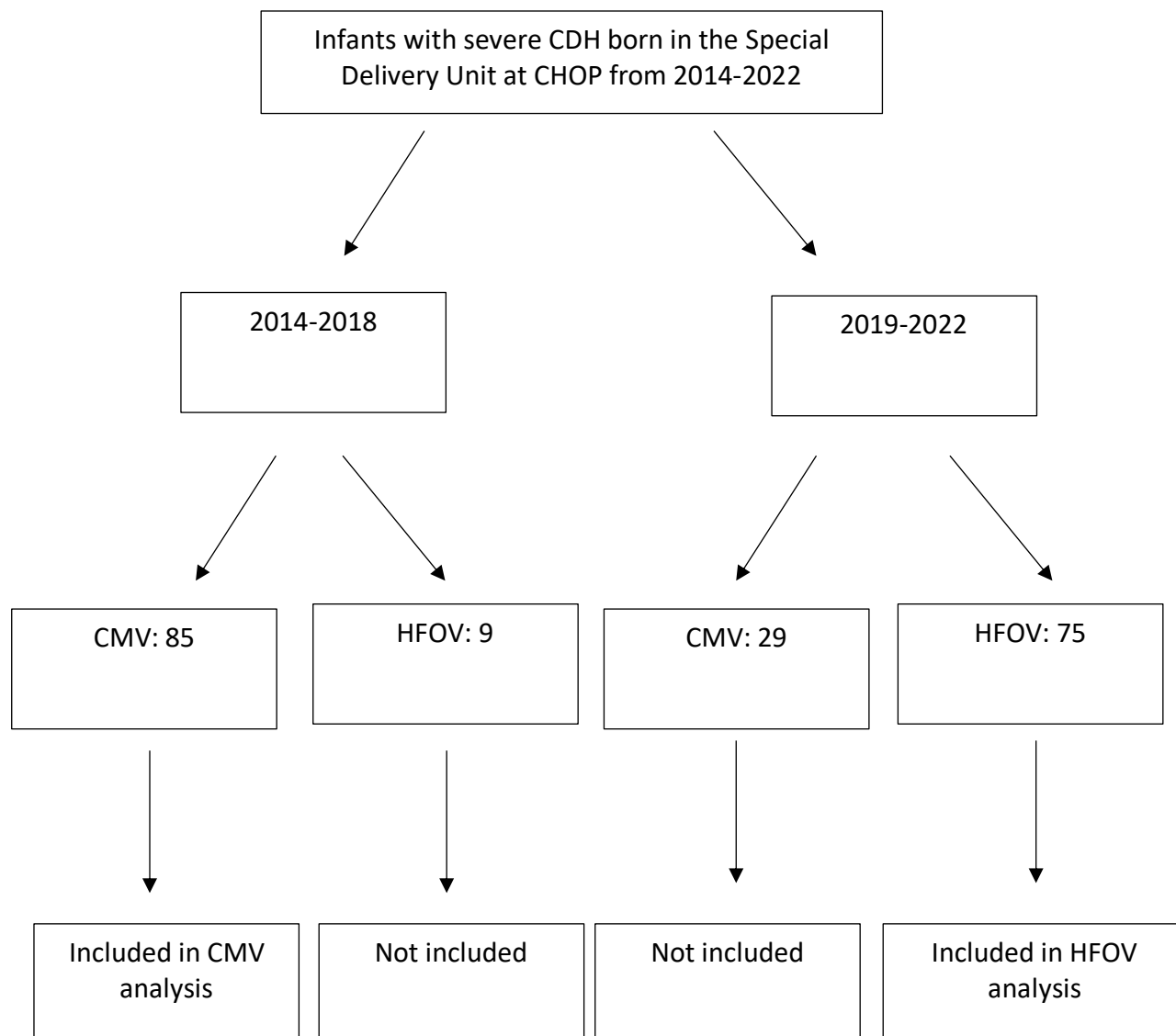

Supplemental Figure 1. Flow diagram of cohort based on initial ventilator mode.

Supplement: Supplementary file 1 — Supplemental Figure 1. Flow diagram of cohort based on initial ventilator mode. [file 41372_2024_2024_MOESM1_ESM.pdf]
