# Supplementary material for: Association between initial ventilation mode and hospital outcomes for severe congenital diaphragmatic hernia
Source: J Perinatol. 2024 Jun 28;44(9):1353–8. doi: 10.1038/s41372-024-02024-z (PMC11379620; doi:10.1038/s41372-024-02024-z)
Supplement: Supplementary file 2 — Supplemental Table 1. Blood Gas Values for the First 48 Hours of Life [file 41372_2024_2024_MOESM2_ESM.docx]

| **Supplemental Table 1. Blood Gas Values for the First 48 Hours of Life** | | | |
| --- | --- | --- | --- |
|  | **Median (IQR)** | |  |
|  | **CMV (n=85)** | **HFOV (n=75)** | **p-value** |
| pH at 1 hour of life | 7.02 (6.90, 7.15) | 7.18 (7.03, 7.24) | **<0.001** |
| CO_2_ at 1 hour of life | 87.5 (66.8, 105.0) | 62.0 (46.0, 82.0) | **<0.001** |
| pH at 6 hours of life | 7.30 (7.19, 7.36) | 7.37 (7.28, 7.43) | **< 0.001** |
| CO_2_ at 6 hours of life | 49.8 (40.7, 61.8) | 38.6 (33.5, 48.3) | **< 0.001** |
| pH at 12 hours of life | 7.32 (7.24, 7.38) | 7.35 (7.28, 7.40) | 0.10 |
| CO_2_ at 12 hours of life | 45.4 (38.8, 57.0) | 41.6 (36.4, 48.7) | **0.05** |
| pH at 18 hours of life | 7.30 (7.24, 7.36) | 7.33 (7.30, 7.38) | **0.02** |
| CO_2_ at 18 hours of life | 45.6 (40.9, 56.5) | 44.0 (39.5, 51.1) | 0.11 |
| pH at 24 hours of life | 7.32 (7.28, 7.37) | 7.32 (7.29, 7.37) | 0.57 |
| CO_2_ at 24 hours of life | 44.5 (38.5 52.3) | 43.6  (37.7, 52.1) | 0.22 |
| pH at 48 hours of life | 7.32 (7.29, 7.36) | 7.33 (7.30, 7.37) | **0.04** |
| CO_2_ at 48 hours of life | 46.2 (42.5, 53.0) | 45.1 (38.7, 52.3) | 0.37 |
| CMV=conventional mechanical ventilation, CO_2_=carbon dioxide, HFOV= High Frequency Oscillatory Ventilation, IQR= Interquartile | | | |
