# Supplementary material for: Association between initial ventilation mode and hospital outcomes for severe congenital diaphragmatic hernia
Source: J Perinatol. 2024 Jun 28;44(9):1353–8. doi: 10.1038/s41372-024-02024-z (PMC11379620; doi:10.1038/s41372-024-02024-z)
Supplement: Supplementary file 3 — Supplemental Table 2. Vasoactive Use and Pulmonary Hypertension Treatment [file 41372_2024_2024_MOESM3_ESM.docx]

| **Supplemental Table 2. Vasoactive Use and Pulmonary Hypertension Treatment** | | | |
| --- | --- | --- | --- |
|  | **N(%)** | |  |
|  | **CMV  (n=85)** | **HFOV  (n=75)** | **p-value** |
| **iNO in first 48 hours of life** | 59 (69.4%) | 49 (65.3%) | 0.70 |
| **iNO anytime during hospitalization** | 73 (85.9%) | 65 (86.7%) | >0.99 |
| **Dopamine infusion in first 48 hours of life** | 67 (78.7%) | 63 (84.0%) | 0.53 |
| **Dopamine infusion anytime during hospitalization** | 74 (87.1%) | 70 (93.3%) | 0.29 |
| **Epinephrine infusion in first 48 hours of life** | 1 (1.2%) | 26 (34.7%) | **<0.001** |
| **Epinephrine infusion anytime during hospitalization** | 9 (10.6%) | 40 (53.3%) | **<0.001** |
| **Alprostadil infusion in first 48 hours of life** | 21 (24.7%) | 34 (45.3%) | **0.01** |
| **Alprostadil infusion anytime during hospitalization** | 46 (54.15) | 51 (68.0%) | 0.10 |
| **Sildenafil at discharge** | 18 (21.2%) | 13 (17.3%) | 0.74 |
| **Treprostinil at discharge** | 3 (3.5%) | 5 (6.7%) | 0.47 |
| **Any Pulmonary Hypertension Medications at Discharge** | 18 (21.2%) | 14 (18.7%) | 0.91 |
| CMV=Conventional Mechanical Ventilation, HFOV= High Frequency Oscillatory Ventilation, iNO=Inhaled Nitric Oxide | | | |
